# Systemic treatments for radioiodine-refractory thyroid cancers

**DOI:** 10.3389/fendo.2024.1346476

**Published:** 2024-10-15

**Authors:** Piaohong Chen, Yu Yao, Huiwen Tan, Jianwei Li

**Affiliations:** Division of Endocrinology and Metabolism, West China Hospital of Sichuan University, Chengdu, China

**Keywords:** radioiodine-refractory thyroid cancers, sodium/iodide symporter, mitogen-activated protein kinase, phosphatidylinositol-3-hydroxykinase, TERTp, tyrosine kinase inhibitors, systemic treatments

## Abstract

Differentiated thyroid cancers (DTCs) constitute the primary histological subtype within thyroid cancer. Due to DTCs’ distinctive radioiodine (RAI) uptake mechanism, standard treatment involving surgery, with or without adjunctive therapy using RAI and levothyroxine inhibition, typically yields favorable prognoses for the majority of patients with DTCs. However, this favorable outcome does not extend to individuals with decreased RAI uptake, termed radioiodine-refractory thyroid cancers (RAI-RTCs). Recent research has revealed that the genetic mutations and gene rearrangements affecting sites such as *RTKs*, *RAS, BRAF* and *TERTp* lead to structural and functional abnormalities in encoded proteins. These abnormalities aberrantly activate signaling pathways like the mitogen-activated protein kinase (MAPK) and phosphatidylinositol-3-hydroxykinase (PI3K) signaling pathways, resulting in thyroid cells dedifferentiation, sodium/iodide symporter (NIS) dysfunction, and consequent the RAI-refractory nature of DTCs. Targeted therapy tailored to mutations presents a promising avenue for the treatment of RAI-RTCs. Lenvatinib and sorafenib, multi-kinase inhibitors, represent the standard first-line systemic treatment options, while cabozantinib is the standard second-line treatment option, for this purpose. Furthermore, ongoing clinical trials are exploring selective kinase inhibitors, immune checkpoint inhibitors, and combination therapies. Notably, numerous clinical trials have demonstrated that selective kinase inhibitors like BRAF, MEK and mTOR inhibitors can restore RAI uptake in tumor cells. However, further validation through multicenter, large-sample, double-blinded randomized controlled trials are essential. Enhanced treatment strategies and innovative therapies are expected to benefit a broader spectrum of patients as these advancements progress.

## 1 Introduction

Thyroid cancer is the most prevalent malignancy within endocrine system (1). According to data from GLOBOCAN 2020 by the International Agency for Research on Cancer, the year 2020 witnessed around 586,000 new cases of thyroid cancer globally, ranking it ninth among all cancers (2). The three encompassed pathological types are differentiated thyroid cancers (DTCs), medullary cancers and anaplastic cancers, with DTCs comprising about 90% of all thyroid cancers (3). The standard treatment for DTCs involves surgery often coupled with radioiodine (RAI) therapy and thyroid stimulating hormone (TSH) suppression. At the same time, more and more studies show that in addition to the above standard treatment for thyroid cancer, active surveillance is also an important strategy (4–6). Most patients with DTCs exhibit a favorable prognosis with low mortality (7). However, a subset of patients with DTCs shows resistance to RAI treatment, leading to disease progression post-treatment (8). These cases, constituting 5% to 15% of DTCs and 50% of metastatic DTCs (7, 9, 10), are termed as radioiodine-refractory thyroid cancers (RAI-RTCs) (11), displaying 5-year disease-specific survival rates of 60% to 70% (12) with a 10-year survival rate of 10% (13). Current treatment options for RAI-RTCs encompass targeted therapy utilizing tyrosine kinase inhibitors (TKIs), immunotherapy, cytotoxic chemotherapy and active surveillance (14). Among these, targeted therapy, particularly with sorafenib, lenvatinib and cabozantinib approved by the Food and Drug Administration (FDA) for RAI-RTCs treatment (15–18), emerges as a relatively established option. However, these agents offer only limited improvement in prognosis. With ongoing research into the pathogenesis of RAI-RTCs, patients now have access to new treatment avenues. This review aims to outline the pathogenesis of RAI-RTCs, novel therapies, particularly advancements in targeted tyrosine kinase inhibitor (TKI) therapies, and the status of clinical trials (completed and ongoing).

## 2 Review

### 2.1 Definition of RAI-RTCs

In 2006, Durante C et al. published a study of the long-term outcome of 444 patients with distant metastases of DTC, which showed that RAI uptake of the lesions in some DTC patients might gradually decrease or even disappear as the disease progressed, leading to limitations of RAI therapy (13). RAI-RTCs have gradually been recognized by researchers since then, but its definition has undergone evolution over time and is still controversial so far. It was not until September 2010, at the 14th International Thyroid Congress, that the definition of RAI-RTCs was initially proposed. RAI-RTCs were characterized by the absence of RAI uptake in one lesion or the lack of clinical evidence indicating additional benefits from RAI therapy (11). In 2015, the American Thyroid Association (ATA) broadened the scope of RAI-RTCs in the Guidelines for the Diagnosis and Treatment of Thyroid Nodules and Differentiated Thyroid Cancer in Adults, stating four manifestations: (i) the malignant/metastatic tissue does not ever concentrate RAI, (ii) the tumor tissue loses the ability to concentrate RAI after previous evidence of RAI-avid disease, (iii) RAI is concentrated in some lesions but not in others; and (iv) metastatic disease progresses despite significant concentration of RAI (3). But the precise definition of “ significant concentration of RAI “ was not specified. About one year later, the consensus for the management of advanced RAI-RTCs was issued by the Spanish Endocrine Society Thyroid Cancer Working Group and the Spanish Rare Cancer Working Group in 2016, recommending that lesions exhibiting high 18-F-deoxyglucose uptake on positron emission tomography/computed tomography (PET/CT) and total cumulative doses of RAI over 22.2GBq (600 mCi) could also be considered as diagnostic criteria for RAI-RTCs (19). Since many confounding factors in the likelihood appraisal and decision making about further RAI therapy, for example technique issues, standardization of radioactive iodine imaging and other limitations, were not given full consideration, the 2015 ATA guidelines were met with disagreement by extended nuclear medicine community. The European Association of Nuclear Medicine (EANM), the Society of Nuclear Medicine and Molecular Imaging (SNMMI), ATA and the European Thyroid Association (ETA) had an interactive meeting in Martinique thereafter in January 2018 with eight countries represented. A set of nine principles (Martinique Principles) were agreed on and published in 2019 (20). It was pointed out that characteristics used to classify patients as RAI refractory should be used to risk stratify patients and not necessarily as definitive criteria to mandate whether RAI therapy should be recommended. Five common clinical scenarios were summarized to be suggestive of the possibility of RAI-RTCs rather than absolute indicators, including: 1) no RAI uptake is present on a diagnostic RAI scan; 2) no RAI uptake is present on a RAI scan performed several days after RAI therapy; 3) RAI uptake is only present in some but not other tumor foci; 4) DTC metastasis(es) progress despite RAI uptake; 5) DTC metastasis(es) progress despite a cumulative RAI activity of 22.2GBq (600mCi). After Martinique meeting, ETA detailed the requirement for the assessment of RAI-RTCs. It was recommended that SPECT-CT be performed after high-activity RAI with preparation of high TSH and a diet with low iodine content; progression be defined as radiological progression, according to the response evaluation criteria in solid tumors (RECIST) 1.1) criteria, within a clinically relevant time frame, which is usually considered 6-12 months (21). In summary, no current definition, classification, criterion, or clinical scenario is an absolute indicator to label a patient as RAI refractory, but they convey the likelihood that a tumor will be refractory to additional RAI therapy. RAI-refractory criteria will continue to evolve, when confounding limitations and technical issues are addressed, techniques for radioactive iodine imaging are optimized and standardized, and the effectiveness of RAI therapy is enhanced by re-differentiation therapies (20).

### 2.2 Pathogenesis of RAI-RTCs

**Figure 1:**
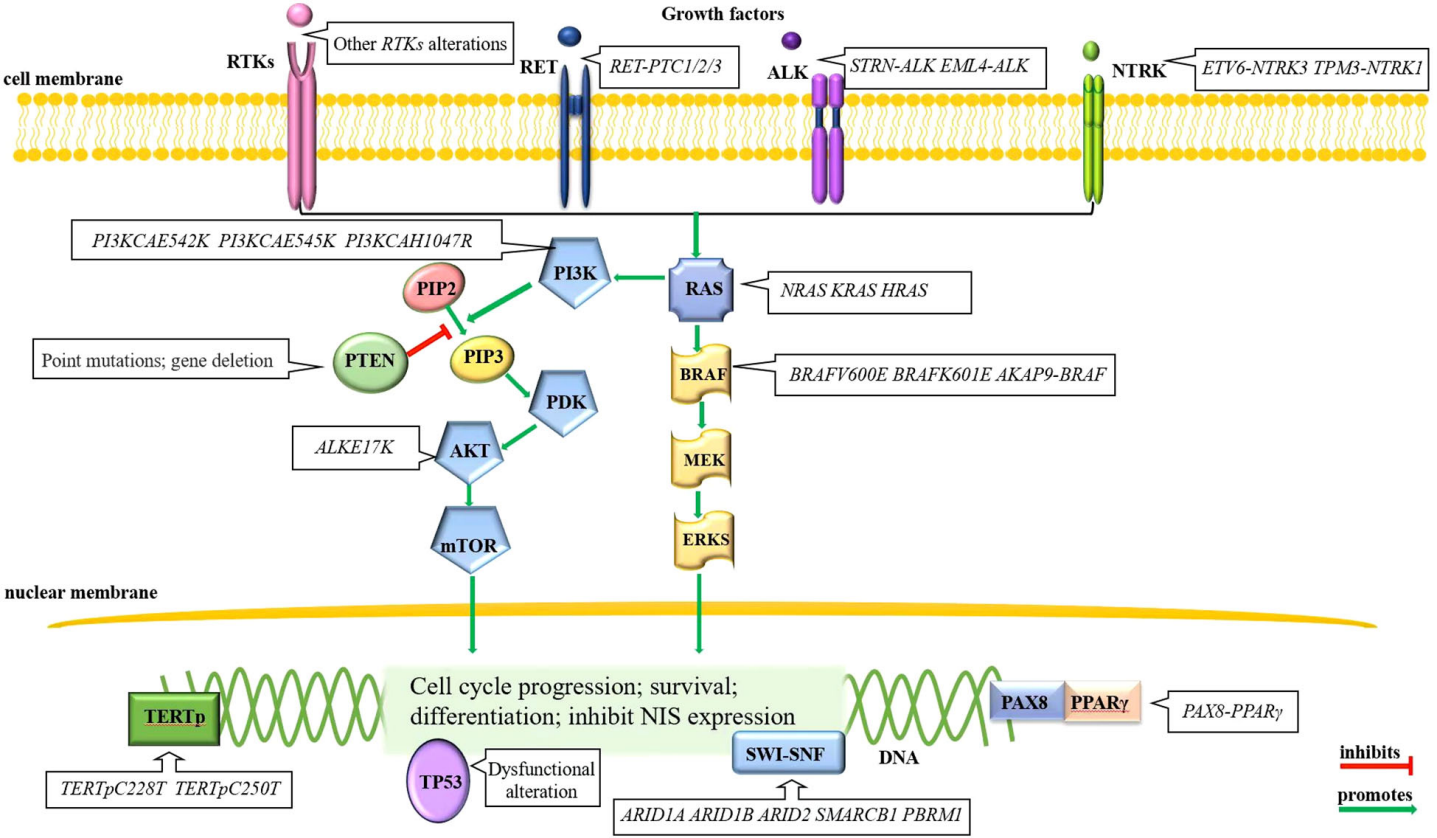
Molecular alterations of RAI-RTCs. Mutually exclusive *BRAF* and *RAS* alterations in the MAPK signaling pathway and gene alterations in *RTKs*, primarily *RET*, are often the initial events of thyroid cancer. Other mutational events, for example, the gene alterations in PI3K signaling pathway, disruption of p53 functions, re-express *TERT*, perturb the SWI-SNF chromatin remodeling complex, etc., drive disease progression (28).

The normal thyroid follicular cell membrane contains the sodium/iodide symporter (NIS), which actively transport two sodium ions and one iodide ion into the cytoplasm simultaneously (22). RAI is absorbed into thyroid tumor cells via NIS, releasing β rays that effectively destroy residual thyroid cancer cells within a range of 2.4 mm (23, 24), thereby playing an important role in the treatment of thyroid cancer. In general, lower *NIS* expression correlates with poorer differentiation of thyroid tumor cells, leading to less RAI uptake and ultimately the development of RAI-RTCs. Therefore, many thyroid-cancer-associated alterations are also playing a role in the development of RAI-RTCs. The pathogenesis process of RAI-RTCs is intricately associated with abnormal activation of the MAPK and/or PI3K signaling pathway, disruption of p53 functions, re-expression of telomerase reverse transcriptase (TERT), perturbation of the SWI-SNF chromatin remodeling complex and some rare genetic alterations (25–28). A brief description can be seen in **Figure 1**.

#### 2.2.1 Activation of MAPK and PI3K signaling pathway

##### 2.2.1.1 Genetic alterations activating both MAPK and PI3K signaling pathways

*RAS* mutations are early events in thyroid tumorigenesis, include three mutations (*KRAS*, *HRAS*, and *NRAS*), with the mutant in codon 61 of *NRAS* being the most common variant (29, 30). The RAS oncoprotein, as a common effector of the PI3K and MAPK signaling pathways, prevalently affects the latter signaling pathway (31). The mutated RAS protein is locked into guanosine triphosphate (GTP), persistently activating the PI3K pathway, which ultimately promotes tumor progression (32).

The *RTKs* genes encode cell-membrane-located kinases that are stimulated by insulin/insulin-like growth factor-1 (IGF-1), epidermal growth factor (EGF) (33), vascular endothelial growth factor (VEGF) and other cytokines (27) and then signal to downstream pathways including MAPK and PI3K signaling pathways. Mutations of *RTKs* genes can cause increased *RTKs* genes transcription, RTKs proteins mislocalization, aberrant RTKs fusion proteins, enhanced affinity for cytokines, cascade activation of the downstream MAPK and PI3K signaling pathways (34, 35), and thereby initiated a subset of thyroid cancers. *RTKs* genetic alterations are mainly gene rearrangement, which occur in proto-oncogene c-ret protein (*RET*), anaplastic lymphoma kinase (*ALK*), and neurotrophic tyrosine kinase receptor (*NTRK*). The *RET* rearrangement with coiled-coil domain-containing gene 6 (*CCDC6*), nuclear receptor coactivator 4 (*NCOA4*) and protein kinase cAMP-dependent type I regulatory subunit alpha (*PRKAR1A*) makes fusion gene *RET-PTC1/2/3* (36), which is common in DTCs (37); the rearrangement of *ALK* and the striatin gene or *EML4*, results in the new fusion genes *STRN-ALK* (38) and *EML4-ALK* (39), respectively; the *NTRK* rearrangement with *ETS* variant gene 6 and α-tropomyosin (*TPM3*) forms the new fusion genes *ETV6-NTRK3* (40) and *TPM3-NTRK1* (41), respectively. Other rare genetic alterations, including gene copy number gain and missense mutations, occur in *PDGFR*, *VEGFR*, *c-KIT*, *FGFR* and *FLT3* (31, 42–44).

It is worth noting that *ALK* fusion and *NTRK* fusion are rather rare in adult thyroid cancers (45, 46). Fusions involving the gene *RET* followed by *NTRK* and *ALK*, are the most prevalent rearrangements found in pediatric papillary thyroid carcinoma (PTC) (47) and have the highest association with invasive disease, particularly in cases of *RET* fusion (48). *RET* fusion genes are three times more frequently in pediatric than in adult patients. A total of 20 types of *RET* fusions have been identified, including *CCDC6*, *NCOA4, RUFY2, AFAP1L2* and *PRKAR1A*, among others (48). Regarding *NTRK* fusion genes, the *ETV6-NTRK3* fusion is most common, followed by *TPR-NTRK1* and then other less frequent fusion patterns with both *NTRK3* and *NTRK1* (49). *ALK* fusion is rare in pediatric PTC, the predominant type identified is STRN-ALK (50).

##### 2.2.1.2 Genetic alterations activating MAPK signaling pathway

*BRAF* mutations are early events in thyroid tumorigenesis (51), including *BRAFV600E* (52) and more rarely, *BRAFK601E* (52, 53). As the predominant form of mutation, the prevalence of *BRAFV600E* is lower in pediatric (especially ≤10 years old) PTC than in adult PTC (47). *BRAF* gene rearrangement (*AKAP9-BRAF*) is more common in radiation-related PTC but has also been reported in poorly differentiated thyroid cancers and anaplastic thyroid cancers (53, 54). It is noteworthy that *BRAF* mutations and *RAS* mutations occurs mutually exclusive.

##### 2.2.1.3 genetic alterations activating PI3K signaling pathway

Thyroid-cancer-associated genetic alterations in the PI3K signaling pathway mainly occur in three categories of genes: genes encoding phosphatidylinositol-4,5-bisphosphate 3-kinase (*PIK3CA*) α catalytic subunit, the serine-threonine protein kinase *AKT*, and phosphatase and tensin homolog phosphatase (*PTEN*). Genetic alterations of *PIK3CA* and *AKT* include point mutations and copy number gains, both are late events in thyroid tumorigenesis (55). Missense mutations of *PIK3CA* take place in exons 9 and 20 (E542K, E545K and H1047R) and are less frequent than copy number gains occurring at chromosome site 3q26.3 (56). These events increase PIK3CA protein expression, yet their tumorigenic role is not well defined. The *AKT* mutation is the E17K substitution, and this mutation can inhibit the apoptosis of thyroid cancer cells (55). It should be noted that unlike the first two oncogenes, *PTEN*, as a tumor suppressor gene (42), promotes *NIS* expression (57) and inhibits the PI3K signaling pathway (42). Genetic alterations of *PTEN* in thyroid cancer include point mutations, heterozygous deletion, whole gene deletion and epigenetic modification (58). The genetic alterations silence *PTEN* and activate the PI3K signaling pathway, resulting in enhanced tumor proliferation and invasion (32, 58).

##### 2.2.1.4 Downstream changes leading to cell dedifferentiation

The abnormal activation of the MAPK signaling pathway can promote the expression of tumor microenvironment (TME)-related genes (59–62) and impair the expression of genes that are required for normal thyroid function (63), which consequently leads to tumor enlargement and distant metastasis (34, 35). The physiological functions of PI3K signaling pathway activation facilitate cell metabolism, growth, proliferation and survival. Aberrant activation of PI3K signaling pathway can aid the uninhibited growth of cancer cells by increasing protein synthesis (64) and by interacting with unrestricted MAPK signaling pathway in thyroid tumor progression.

##### 2.2.1.5 Downstream changes leading to regulation of NIS expression

**Figure 2:**
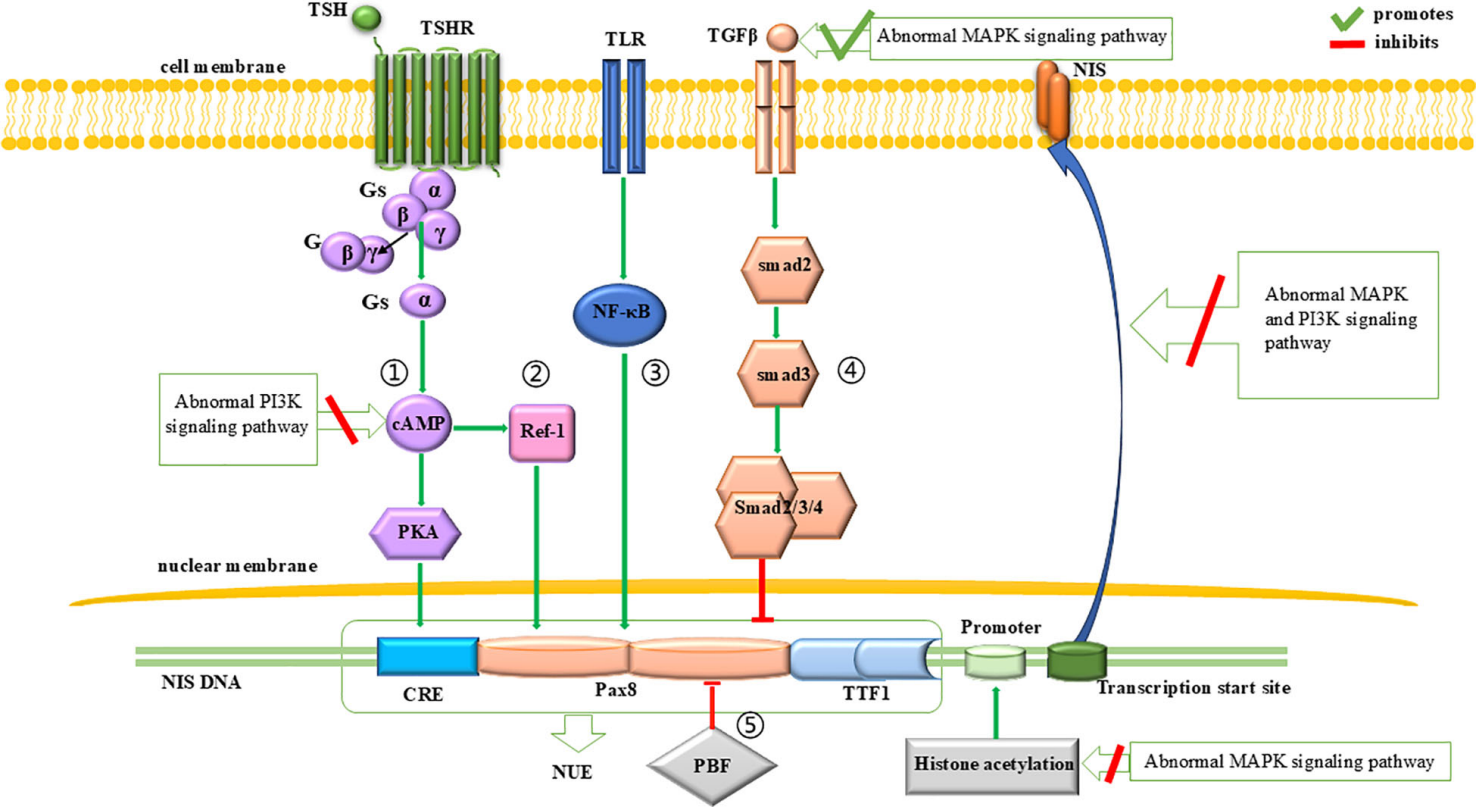
Regulation of *NIS* expression. The expression of *NIS* is influenced by the PI3K and MAPK signaling pathways. At the transcriptional level, abnormal MAPK signaling pathway initially reduces NIS expression by inhibiting histone acetylation at the promoter region. Subsequently, it downregulates NIS transcription by promoting the inhibitory effects of the TGFβ-Smad signaling pathway on NUE. Additionally, abnormal PI3K signaling pathway can inhibit the promotion effect of TSH-dependent NIS expression pathway on NUE, thereby leading to a downregulation of NIS expression. At the translational level, both abnormal PI3K and MAPK signaling pathways can impair the proper localization of NIS to the cell membrane.

Beside of cell dedifferentiation and proliferation, impaired function or decreased expression of *NIS* is another major mechanism contributing to the RAI-refractory nature of thyroid cancer. The expression of *NIS* is regulated at both the transcription and posttranscription levels. The abnormal activation of MAPK and PI3K signaling pathway can inhibit NIS expression by promoting TGFβ-Smad signaling pathway and interfering the proximal promoter region of *NIS* for the former (65), and by inhibiting TSH-dependent NIS expression pathway for the latter (33, 66). The abnormal activation of both signaling pathways can also interfere with the correct localization of NIS protein to the cell membrane (67). A brief description can be seen in **Figure 2**.

###### 2.2.1.5.1 Regulation of *NIS* expression at the level of transcription

*NIS* expression is regulated by two regions: the proximal promoter (68) and the *NIS* upstream enhancer (NUE) (69).

For the proximal promoter, the abnormal activation of the MAPK signaling pathway can downregulate the histone acetylation within this region, leading to the silencing of NIS expression (42, 70–73).

At the NUE, regulation of *NIS* transcription involves a cAMP-response element (CRE)-like sites (69, 74–76) and two paired box 8 (Pax8) binding sites (69). As for the CRE-like sites, their cAMP- and PKA-dependent phosphorylation activates NUE and promote *NIS* expression (76, 77). Since cAMP is produced by the action of extracellular thyroid stimulating hormone (TSH) (27), this pathway is also known as the TSH-dependent NIS expression pathway (**Figure 2**, pathway 1). This pathway can be inhibited by an abnormally activated PI3K signaling pathway (33, 66). As for the Pax8 binding sites, Pax8 binding to NUE can activate NUE and promote *NIS* expression (78, 79). This process is regulated by four signaling pathways: (1) A protein kinase A (PKA)-independent pathway, where cAMP promotes the binding of Pax8 to NUE through redox effector factor-1 (Ref-1) (75, 80–82) (**Figure 2**, pathway 2); (2) The Toll-like receptor (TLR)-NF-κB pathway, in which TLRs are stimulated by extracellular signals and promote the binding of Pax8 to NUE through NF-κB (83, 84) (**Figure 2**, pathway 3); (3) The TGFβ-Smad signaling pathway, in which Smad3 activation by TGFβ can inhibit the binding of Pax8 to NUE (59, 80) (**Figure 2**, pathway 4), and it is notable that the abnormal activation of the MAPK signaling pathway can promote TGFβ secretion and inhibit *NIS* expression (65); (4) the pituitary tumor-transforming gene-1 product (PTTG1)-binding factor (PBF) complex, which can interfere with the binding of Pax8 to NUE (85–87) (**Figure 2**, pathway 5).

###### 2.2.1.5.2 Regulation of *NIS* expression at the level of posttranscription

Abnormal modification of the NIS protein or its incorrect localization to the cell membrane leads to NIS dysfunction. NIS protein modifications (including phosphorylation and glycosylation) and cell membrane localization (88, 89) are regulated not only by TSH (89–91) but also by the MAPK and PI3K signaling pathways. Previous studies have shown that abnormal activation of the MAPK and PI3K signaling pathways inhibit the correct localization of NIS protein to the cell membrane (67).

#### 2.2.2 Disruption of p53 functions

The p53 is a tumor suppressor encoded by *TP53* that facilitates cell cycle control, DNA repair and apoptosis in response to various cellular stresses, thereby quenching the growth and proliferation of abnormal cells (92, 93). Genetic inactivation of p53 enable mutant tumor cells to circumvent these checkpoints (94). Indeed, p53 deficiency in association with activating mutations of oncogenes, such as *RAS* and *BRAF*, accounts for the high proliferation rate and increased aggressiveness of the more aggressive forms of thyroid cancer (95).

#### 2.2.3 Re-expression of TERT

*TERT* is important for maintaining chromosomal integrity and genome stability (96). In most somatic cells, telomeres shorten with cell division, and when their length reaches a critical point, cells enter senescence or apoptosis (96).However, in thyroid cancer cells, an activating mutation in the *TERT* promoter (*TERTp*) prevents telomere shortening, allowing tumor cells to continue dividing and proliferating (97), thereby driving disease progression. The common *TERTp* mutations in thyroid cancer are two mutually exclusive mutations, C228T and C250T (98). These mutations form a consensus binding site for the E-twenty-six (ETS) transcription factor in the *TERTp* region, increasing its transcriptional activity (98). *TERTp* mutations, as a late event in tumor progression (55, 98), can also occur simultaneously with *BRAF* or *RAS* mutations in poorly differentiated and anaplastic thyroid cancers (99, 100), enhancing tumor aggressiveness (100–105). Therefore, they can be served as an early predictor of RAI-RTCs (106). Recently, some mouse model studies have shown that *TERT* reactivation can accelerate progression of *BRAF*-driven thyroid tumors via non-telomeric effects such as cytokine and PI3K signaling (107, 108). This association of *TERT* and PI3K signaling pathway may shed some light on the role of *TERT* in the pathogenesis of thyroid cancer as well as RAI-RTCs.

#### 2.2.4 Perturbation of the SWI-SNF chromatin remodeling complex

The function of SWI-SNF complexes is to reconfigure chromatin, thereby determining the expression of certain genetic programs. Dysfunction of SWI-SNF can induce stem cell-likeness (109). Genetic alterations disrupt genes that encode members of the SWI-SNF chromatin remodeling complex (for example, *ARID1A, ARID1B, ARID2, SMARCB1* or *PBRM1*) occur in 6% of PDTCs and 36% of ATCs (55). Among those, thyroid cancers with *BRAFV600E* and SWI-SNF impairment were locked in a dedifferentiated transcriptional state that could not be reversed by MAPK signaling pathway inhibition (110), which would discourage the use of redifferentiation strategies.

#### 2.2.5 Genetic alterations in other loci

Changes in the WNT/β-catenin signaling pathway (111), histone deacetylase (HDAC) isoforms (112), aberrant gene methylation (113, 114), peroxisome proliferator activated receptor gamma (*PPAR-γ*) rearrangement with *PAX8 (* 115), and imbalance of ncRNAs (116) have also been observed in RAI-RTCs and take part in its occurrence and development.

### 2.3 Timing of systemic therapy for RAI-RTCs

The initiation of systemic therapy for RAI-RTCs should be done with caution (11). First of all, in some patients the condition can remain stable for many years and their life expectancy can be as long as several decades. In addition, drug-related adverse reactions may lead to a decreased quality of life. Finally, local treatment such as stereotactic radiotherapy and thermal ablation would be preferred for local advanced lesions (3), making immediate systemic therapy upon diagnosis of RAI-RTCs unnecessary. Some studies have suggested that targeted therapy be initiated when the tumor doubling time (VDT) is less than 6 months (117). Guidelines for the Diagnosis and Treatment of Thyroid Nodules and Differentiated Thyroid Cancer in Adults issued by ATA in 2015 recommended initiating TKI therapy for RAI-RTCs if the disease is metastatic, rapidly progressive, symptomatic, and/or imminently threatening disease not otherwise amenable to local control using other approaches (3). As TKIs are accompanied by side effects, ETA agree that treatment with TKIs should only be considered in patients with progressive RAI-RTC, with considerable tumor load and when, according to a multidisciplinary group of experts, refraining from treatment with MKIs would lead to considerable harm/clinical complications within the near future. Before starting MKIs, local treatments should be considered (21). European Society for Medical Oncology (ESMO) recommended initiating TKI therapy in patients with advanced/metastatic DTCs (118), and the specific evaluation criteria should be based on symptoms, tumor burden, the Eastern Cooperative Oncology Group performance status (ECOG PS), lesion characteristics (e.g. paratracheal location or other features likely to cause symptoms) and disease progression (119). In summary, decisions should be made after close monitoring and assessment of the disease. The best time to start TKI therapy for asymptomatic RAI-RTC patients was explored in the international, prospective, multicenter clinical study RIFTOS MKI (registration number NCT02303444), which recruited 647 asymptomatic RAI-RTCs patients (120). Unfortunately, the latest findings from RIFTOS MKI cannot answer the best time to initiate TKI therapy for asymptomatic RAI-RTC patients yet because of the slow accrual of events, with only 13 US patients receiving MKI treatment at study entry (14). Further research is needed to identify the best time to initiate systemic therapy for RAI-RTCs.

### 2.4 Systemic therapies for RAI-RTCs

Most of systemic therapies for RAI-RTCs are targeted therapies for specific mutated proteins while immunotherapy and cytotoxic chemotherapy are also included. Targeted therapies mainly include MKIs and selective kinase inhibitors. Among the MKIs, sorafenib and lenvatinib are currently recommended as standard regimens. Selective kinase inhibitors that can increase the RAI uptake of tumor cells have become a research hotspot in recent years. Although the efficacy of immune checkpoint inhibitor (ICI) monotherapy is limited, there are many combinations of ICIs with targeted therapy or chemoradiotherapy in clinical trials. Cytotoxic chemotherapy is only used as a complementary treatment to the abovementioned options because of its limited clinical benefit (3).

#### 2.4.1 MKIs

In addition to VEGFR, PDGFR, FGFR, RET and c-KIT, which all belong to RTKs, the targets of MKIs also include BRAF and RAS. Representative drugs include sorafenib, lenvatinib, motesanib, pazopanib and sunitinib, among which sorafenib and lenvatinib have been approved by the US FDA for the clinical treatment of RAI-RTCs. The other drugs have been shown to be effective in preliminary clinical trials, yet have not been approved to include RAI-RTCs in their indications for their lack of multicenter large-sample trial data.

Sorafenib was originally developed by Bayer (Leverkusen, Germany), and its main targets are VEGFR 1-3, RET, RAF (including BRAF and C-Raf), PDGFR-β, c-KIT and FLT3 (121). In an earlier phase II single-arm clinical trial that included 31 patients with advanced RAI-RTCs, the median progression-free survival (PFS) after treatment was 18 months, and the median overall survival (OS) was 34.5 months (122). More reliable data were obtained in a multicenter, double-blinded, randomized controlled phase III clinical trial (DECISION; ClinicalTrials.gov number, NCT00984282) in 2012, with 417 patients included. The median PFS was significantly increased in the sorafenib group (n=207) compared with that in the placebo group (n=209) (10.8 months *vs*. 5.8 months) (123). Therefore, sorafenib was approved by the US FDA and the European Medicines Agency in November 2013 for the treatment of advanced RAI-RTCs with a recommended starting dose of 800 mg/day in divided doses. The common adverse effects are palm-plantar swelling, diarrhea, hair loss, rash and scaling, which primarily occur during the first 6 cycles of treatment and may gradually get tolerated as the course of treatment prolongs. However, these adverse events resulted in discontinuation of treatment in 66% of patients, dose reduction in 64% of patients, and permanent discontinuation in 18% of patients (124).

Lenvatinib targets VEGFR 1-3, FGFR 1-4, PDGFR-α, RET and c-KIT (125). Previous studies have suggested that its antitumor effect is mainly directed against the microvascular environment of VEGFR and FGFR rather than against tumor cell proliferation or a specific phase of the cell cycle (126, 127). The efficacy of lenvatinib has been demonstrated in a randomized, double-blinded, multicenter phase III clinical trial (SELECT; ClinicalTrials.gov number, NCT01321554). A total of 392 patients with advanced RAI-RTCs were recruited in this study, and the primary endpoint was PFS. The PFS of the lenvatinib group (n=261) and the placebo group (n=131) were 18.3 and 3.6 months, respectively. The treatment response rate of the Lenvatinib group was 64.8% and four patients had complete responses (CR), while that of the control group was only 1.5% and all responses were partial (128). The US FDA approved lenvatinib as the second drug, after sorafenib, for the treatment of advanced RAI-RTCs in 2015, with a recommended starting dose of 24 mg/day (129). The most common adverse reactions are hypertension, diarrhea, fatigue, nausea, loss of appetite and weight loss. Adverse reactions led to discontinuation in 82.4% of patients, dose reduction in 67.8% and permanent discontinuation in 14% (128). A study exploring the optimal dose of lenvatinib to treat RAI-RTCs has shown that the 24-week objective response rates (ORRs) of the lenvatinib were 57.3% in the 24 mg/day (n=75) group and 40.3% in the 18 mg/day (n=77) group, respectively, with the odds ratio (OR) being 0.5. As for the incidences of adverse reactions, treatment-emergent adverse events (TEAEs)≥3 in each group were 61.3% and 57.1%, respectively. Therefore, the starting dose of 24 mg/day is confirmed and recommended (130) (**Table 1**).

**Table 1:** Clinical trials of MKIs for RAI-RTCs treatment.

Agent	Combination	Study population	Design	Patients	Primary end point	Status	Identifier
**envatinib**	NA	RAI-RTC	Non-Randomized, Open Label	12	RAI treatable rate	Recruiting	NCT04858867
**Lenvatinib**	Pembrolizumab	RAI-RTC	Non-Randomized, Open Label, Phase I	60	CRR	Active, not recruiting	NCT02973997
**Vandetanib**	NA	RAI-RTC	Randomized, Double-Blind, Phase III	243	PFS	Active, not recruiting	NCT01876784
**Cabozantinib**	NA	RAI-RTC	Non-Randomized, Open Label, Phase I	43	Number of Adverse Events	Active, not recruiting	NCT02041260
**Cabozantinib**	Ipilimumab/Nivolumab	RAI-RTC pretreated with anti-VEGFR	Non-Randomized, Open Label, Phase I	24	ORR	Active, not recruiting	NCT03914300
*Imatinib	NA	RAI-RTC	Non-Randomized, Open Label, Phase I	18	Restore iodine uptake	Unknown	NCT03469011

NA, not available; RAI-RTC, radioiodine-refractory thyroid cancers; RAI, radioactive 131 iodine; CRR, complete response rate; PFS, progression-free survival; TTP, time to disease progression; ORR, objective response rate; anti-VEGFR: VEGFR inhibitor. * indicates that the trial evaluated the RAI uptake of the lesion by the intervention.

Cabozantinib, an inhibitor of VEGFR, AXL, MET, and RET, is the second-line treatment option in advanced RAI-RTC patients. The latest results from COSMIC-311 (registration number NCT 03690388) indicated that cabozantinib benefits patients with RAI-RTC, regardless of prior lenvatinib or sorafenib treatments (131). COSMIC-311 is an international, randomized, double-blind trial in which patients with locally advanced or metastatic RAI-RTC that progressed during or following treatment with lenvatinib, sorafenib, or both were treated with either cabozantinib (n = 170) or placebo (n = 88). The median PFS with cabozantinib was 16.6, 5.8, and 7.6 months for those patients who received prior sorafenib only, prior lenvatinib only, or both, respectively, versus 3.2, 1.9, and 1.9 months with placebo; hazard ratios (HRs) 0.13, 0.28, and 0.27, respectively (132). Therefore, cabozantinib has been approved by FDA and Germany as a second-line treatment option in advanced RAI-RTC (133, 134).

Another MKI drug, motesanib, targets VEGFR 1-3, PDGFR, RET and c-KIT (135). In an open-label single-arm phase II clinical trial of 93 patients, 14% had a partial response (PR) and 35% had stable disease (SD) for more than 24 weeks (136). Vandetanib is shown efficacy in a randomized phase 2 trial in 145 patients with locally advanced or metastatic differentiated thyroid carcinoma (18). Patients who received vandetanib had longer PFS than did those who received placebo (HR 0·63, 60% CI 0·54-0·74; one-sided p=0·008): median PFS was 11·1 months (95% CI 7·7-14·0) for patients in the vandetanib group and 5·9 months (4·0-8·9) for patients in the placebo group. And vandetanib has been approved by the FDA for the treatment of advanced medullary thyroid carcinoma (137). A randomized double-blind phase III clinical trial of vandetanib efficacy in RAI-RTC patients is still ongoing (see **Table 1** for details).

Other MKIs include pazopanib (138), sunitinib (139) and imatinib. Clinical trials, most of which are small sample, single-arm, open-label, single-center phase II studies, have shown that pazopanib (140, 141) and sunitinib (142, 143) have some efficacy in RAI-RTCs patients, which still needs further verification. As for imatinib, a small clinical trial investigating whether it can promote RAI reuptake in RAI-RTCs is ongoing (see **Table 1** for details).

In summary, sorafenib and lenvatinib have been approved by the FDA for the treatment of RAI-RTCs with the dose reduction and treatment discontinuation due to adverse reactions worthy of attention yet. Cabozantinib can be used as a second-line treatment for RAI-RTC that has progressed after sorafenib and Lenvatinib. The other MKIs, such as pazopanib, motesanib and sunitinib, have shown certain clinical efficacy in RAI-RTCs but large sampled multicenter randomized controlled trials are still needed to confirm their efficacy. Off-label use of unapproved MKIs may also be considered if disease progression occurs despite treatment with sorafenib or lenvatinib (144).

#### 2.4.2 Selective kinase inhibitors

There are many types of selective kinase inhibitors including RET inhibitors, BRAF inhibitors, MEK inhibitors, mTOR inhibitors, VEGFR-2 inhibitors and histone deacetylase inhibitors (HDACis). Their therapeutic effect in thyroid cancer, has been confirmed by a number of clinical studies. Notably, BRAF and MEK inhibitors have achieved a major breakthrough in the treatment of RAI-RTCs with the *BRAF^V600E^* mutation, which can cause RAI reuptake in some RAI-RTC lesions carrying *BRAF/RAS* mutations, allowing response to RAI treatment (145).

##### 2.4.2.1 RET-specific inhibitor

The representative drugs of this class are selpercatinib and pralsetinib. Selpercatinib is approved by US FDA for the treatment of thyroid cancer with *RET* gene mutations or fusions (146). In a phase I-II trial(NCT03157128), selpercatinib showed durable efficacy in 19 patients with previously-treated RET fusion-positive thyroid cancer, and the response rate was 79% (95% CI, 54–94) (147). Since *RET* mutation mainly occur in MTC (148), selpercatinib is mainly approved for the treatment of MTC. At present, several clinical trials of selpercatinib for RET-altered thyroid cancer are still ongoing, including the study of whether selpercatinib can increase the uptake of RAI in RET-altered RAI-RTC. The results of these studies are promising (see **Table 2**). Pralsetinib is another strong RET inhibitor. In the ARROW study (NCT03037385), the ORR of pralsetinib in 22 previously treated RET fusion positive thyroid cancer patients was 90.9% (95%CI: 70.8-98.9) (149). In December 2020, the FDA approved pralsetinib for use in patients with RET fusion positive RAT-RTC (150). More studies on pralsetinib for RAI-RTC are shown in **Table 2**.

**Table 2:** Clinical trials of single-target inhibitors in the treatment of RAI-RTCS and other thyroid cancers.

Agent	Combination	Study population	Design	Patients	Primary end point	Status	Identifier
*Larotrectinib	RAI	DTC harboring NTRK Fusions	Non-Randomized, Open Label, Phase II	13	ORR	Recruiting	NCT05783323
**Larotrectinib/Entrectinib**	with or without anti-PD-1	TC pretreated with anti-VEGFR	Non-Randomized, Open Label, Phase IV	200	ORR	Recruiting	NCT06195228
**Dabrafenib**	Lapatinib	BRAF+TC	Non-Randomized, Open Label, Phase I	21	MTD	Active, not recruiting	NCT01947023
**Dabrafenib**	Trametinib	BRAF+TC	Randomized, Open Label, Phase II	53	ORR	Active, not recruiting	NCT01723202
**Dabrafenib**	Trametinib	RAS/BRAF + RAI-RTC	Non-Randomized, Open Label, Phase II	87	ORR	Active, not recruiting	NCT03244956
**Dabrafenib**	Trametinib	BRAF + RAI-RTC	Randomized, Placebo-controlled Double-blind, Phase IV	150	PFS	Recruiting	NCT04940052
*Dabrafenib	Trametinib	RAI-RTC	Non-Randomized, Open Label, Phase II	70	RAI intake rate	Recruiting	NCT04619316
*Vemurafenib	Copanlisib	RAI-RTC	Non-Randomized, Open Label, Phase Ib	22	MTD	Recruiting	NCT04462471
**Selumetinib**	RAI	RAI-RTC	Randomized, Double-Blind, Phase II	60	ORR	Active, not recruiting	NCT02393690
*Trametinib	RAI	RAI-RTC	Non-Randomized, Open Label, Phase II	35	OS, ORR, PFS	Active, not recruiting	NCT02152995
**Everolimus**	Pasireotide	RAI-DTC, MTC	Randomized, Open Label, Phase II	42	CR, PR, OR	Completed	NCT01270321
**Sirolimus**	Cyclophosphamide	RAI-RTC	Non-Randomized, Open Label, Phase II	19	ORR	Recruiting	NCT03099356
**Apatinib**	NA	RAI-RTC	Non-Randomized, Open Label, Phase II	20	DCR, ORR	Completed	NCT02731352
**Apatinib**	NA	RAI-RTC	Randomized, Double-Blind, Phase III	118	PFS	Active, not recruiting	NCT03048877
**Apatinib**	Camrelizumab	RAI-RTC	Non-Randomized, Open Label, Phase II	10	PFS	Recruiting	NCT04560127
**Apatinib**	NA	Advanced/ Metastatic DTC	Non-Randomized, Open Label, Phase II	20	DCR	Unknown	NCT03167385
**Apatinib**	NA	Advanced/ Metastatic DTC	Non-Randomized, Open Label, Phase II	40	ORR	Unknown	NCT03199677
**Selpercatinib**	NA	RET-altered TC	Non-Randomized, Open Label, Phase II	30	ORR	Recruiting	NCT04759911
*Selpercatinib	NA	RET-altered RAI-RTC	Non-Randomized, Open Label, Phase II	30	ORR	Recruiting	NCT05668962
**Selpercatinib/Pralsetinib**	With or without anti-PD-1	TC	Non-Randomized, Parallel Assignment, Single blind, Phase IV	200	ORR	Recruiting	NCT06195228
**Pralsetinib**	NA	RET-altered TC and other solid tumor	Non-Randomized, Parallel Assignment, Open Label, Phase I-II	589	ORR	Active, not recruiting	NCT03037385

TC, thyroid cancer; DTC, differentiated thyroid cancer; MTC, medullary thyroid carcinoma; NA, not available; RAI-RTC, radioiodine-refractory thyroid cancer; DCR, disease control rate; ORR, overall objective response rate; RAI, radioactive 131 iodine, PFS, progression-free survival; MTD, maximum tolerated dose; CR, complete response; PR, partial response; OR, overall response; OS, overall survival; * indicates that the trial evaluated the RAI uptake of the lesion by the intervention.

##### 2.4.2.2 Selective TRK inhibitor

Currently, two drugs have received approval for the treatment of solid tumors with NTRK fusions, larotrectinib and entrectinib.

The efficacy of larotrectinib, a highly selective TRK inhibitor targeting TRKA, TRKB, and TRKC, was examined in phase 1 and 2 clinical trials, in 17 different TRK fusion-positive cancer types, including thyroid cancer. The ORR response reached 75% (151). Based on these studies, larotrectinib received a tissue-agnostic drug approval in patients with TRK fusions. In a pooled analysis from three phase I/II larotrectinib clinical trials, there were 22 patients with TRK fusion-positive DTC treated with larotrectinib, ORR was 86% (152). See **Table 2** for more information.

Entrectinib is another selective inhibitor targeting TRKA, TRKB, TRKC, ALK, and ROS1. Analysis of all phase 1/2 trials evaluating efficacy and safety of entrectinib showed that among 54 adult patients, the ORR was 57%. There were 13 patients with thyroid cancer, 10 patients with PTC, and 3 patients non-PTC, with 1 patient having CR and 6 patients PR, with median duration of response being 13.2 months (153). See **Table 2** for more information.

That both the FDA and EMA approvals for larotrectinib and entrectinib are for patients with metastatic, unresectable solid tumors harboring NTRK fusions in tumor agnostic indication that have no satisfactory treatment options or have progressed on standard-of-care treatment (154).

##### 2.4.2.3 Selective kinase inhibitors of the MAPK signaling pathway

Currently, the main BRAF inhibitors are dabrafenib and vemurafenib, while the MEK inhibitors are selumetinib and trametinib. Studies have shown that selective kinase inhibitors of the MAPK pathway can partially restore RAI uptake in RAI-RTC lesions.

For dabrafenib, a phase I clinical trial of 14 patients with *BRAF^V600E^* -positive RAI-RTCs, showed PR of 29% and SD of 50% (155), and a phase II clinical trial of 10 patients with *BRAF^V600E^*-positive RAI-RTC showed RAI reuptake, with PR of 20% and SD of 40% (156).

For vemurafenib, a phase II clinical trial of 51 patients with *BRAF^V600E^*-positive RAI-RTC showed some efficacy in restoring RAI reuptake (157). Another study suggested that four of 10 *BRAF^V600E^*-positive RAI-RTC patients showed RAI reuptake in lesions (158). The main adverse reactions are rash, weight loss, fatigue and hyperbilirubinemia (127). A clinical trial of the maximum tolerated dose of vemurafenib in combination with a PI3K inhibitor is currently ongoing (detailed in **Table 2**).

For selumetinib, a clinical trial of 20 DTC patients with BRAF mutations or *NRAS* mutations revealed PR of 25% and SD of 15% after RAI treatment (159). To further evaluate whether selumetinib could improve RAI uptake in patients with DTC, AstraZeneca (Cambridge, UK) conducted a multicenter double-blinded phase III randomized controlled trial (NCT01843062) that included a total of 233 patients. The latest results from this study show that although the addition of selumetinib to adjuvant RAI failed to improve the CR rate for DTC patients (160). More information about this study is shown in **Table 2**. Trametinib is another MEK inhibitor. MERAIODE is a multicenter, prospective phase II trial in patients with RAI-RTC, with two independent cohorts: one for BRAFp.V600E patients and one for *RAS* mutated patients (NCT 03244956). In the cohort of RAS mutated patients, the treatment with trametinib is not highly effective for restoring/increasing RAI uptake (161). In the cohort of BRAFp.V600E patients, the designs were similar to the RAS mutated cohort except for treatment consisting of trametinib- dabrafenib, so we present its outcomes in the combined therapy (refer to 4.5). More studies on the efficacy of trametinib in the treatment of RAI-RTC are presented in the part 4.5 and **Table 2**.

##### 2.4.2.4 Selective kinase inhibitors of the PI3K signaling pathway

Drugs that target this signaling pathway include the PI3K inhibitor (buparisib) and the mTOR inhibitors (everolimus, sirolimus and temsirolimus). The sample sizes of clinical trials for these drugs are small (19-43 cases in most trials), therefore the studies with larger sample size are needed to confirm their efficacy in RAI-RTC. Buparisib did not prolong PFS in RAI-RTC patients (162), thus its further study is limited. The efficacy of everolimus has been demonstrated in a study of 28 patients with RAI-RTCs (65% SD), with median PFS and OS being 9 and 18 months, respectively (163). A recent phase II trial of everolimus involving 33 patients with RAI-RTC achieved favorable results (median PFS 12.9 months, 2-year PFS 23.6%) (164).

##### 2.4.2.5 VEGFR-2 inhibitor

The representative drug of this class is apatinib. Two clinical trials with 20 RAI-RTCs patients from mainland China showed that apatinib can reduce serum thyroglobulin levels and tumor volume in patients with RAI-RTCs (165) and obtain a certain efficacy (median PFS: 18.4 months, median OS: 51.6 months) (166). There are currently five ongoing studies evaluating the efficacy of apatinib in RAI-RTC and advanced DTC, one of which is a randomized double-blinded phase III clinical trial with a sample size of more than 100 cases (**Table 2**).

##### 2.4.2.6 HDACis

HDACis can inhibit tumor proliferation, promote tumor differentiation and induce tumor cell apoptosis as well as cell cycle arrest. Valproic acid is a representative drug. Basic studies have shown that HDACis can increase the RAI uptake rate of thyroid cancer cells (167). However, a phase II clinical trial of 13 patients showed that neither RAI uptake nor tumor response in RAI-RTC patients was increased by valproic acid (168). Currently, there is a lack of evidence supporting the efficacy of HDACis in RAI-RTC.

#### 2.4.3 Immunotherapy

Immune checkpoint inhibitors (ICIs) are a new type of antitumor drug which can achieve antitumor goal by blocking the binding of immune checkpoints to their ligands, thereby enhancing the activity of T cells. ICIs mainly include CTLA-4 inhibitors and PD-1/PD-L1 inhibitors, which mainly used in the treatment of melanoma, Hodgkin’s lymphoma and non-small cell lung cancer (NSCLC). But the use of ICIs in thyroid cancer is questioned. On one hand, a large number of immune cells infiltrate DTC tissues, and this is closely related to tumor prognosis (169–173). Tumor patients with high expression of programmed death 1 (PD-1) and programmed death ligand 1 (PD-L1) often have an increased risk of tumor recurrence and shortened disease-free survival (DFS) (174–177). These mechanisms suggest that ICIs should be effective in treating thyroid cancer. On the other hand, the expression of PD-L1 in thyroid cancer fluctuates greatly (6.1% to 82.5%) (178), which creates uncertainty about the efficacy of ICIs to thyroid cancer.

Currently, the clinical application of ICIs for the treatment of malignant tumors includes CTLA-4 inhibitors (ipilimumab), PD-1 inhibitors (pembrolizumab and nivolumab), and PD-L1 inhibitors (durvalumab and atezolizumab). A single-arm study of 22 patients with PD-L1-positive DTC preliminarily explored the efficacy of pembrolizumab to thyroid cancer. The results were two cases of PR (9%) and a median PFS of 7 months (179), suggesting little efficacy of ICIs. Six studies of ICIs combined with targeted therapy or chemoradiotherapy are now in progress and most of them are nondouble-blinded phase II clinical trials with small sample sizes (refer to 4.5, see **Table 3** for details).

**Table 3:** Clinical trials of ICIs and their combination with other drugs in the treatment of thyroid cancer.

Agent	Combination	Study population	Design	Patients	Primary end point	Status	Identifier
**Nivolumab**	Ipilimumab	RAI-RTC, MTC, ATC	Randomized, Open Label, Phase II	53	ORR	Active, not recruiting	NCT03246958
**Pembrolizumab**	Docetaxel	TC/SGT	Non-Randomized, Open Label, Phase II	45	ORR	Recruiting	NCT03360890
**Atezolizumab**	Chemotherapy	Anaplastic and Poorly DTC	Non-Randomized, Open Label, Phase II	50	OS	Recruiting	NCT03181100
**Durvalumab**	RAI	RAI-RTC	Non-Randomized, Open Label, Phase I	11	DLTs	Active, not recruiting	NCT03215095
**Durvalumab**	Tremelimumab	RAI-RTC	Non-Randomized, Open Label, Phase I	46	PFS; OS	Recruiting	NCT03753919
**PDR001**	Dabrafenib/ Trametinib	RAI-RTC	Randomized, Open Label, Phase II	30	ORR	Recruiting	NCT04544111

NA, not available; DTC, differentiated thyroid cancer; MTC, medullary thyroid carcinoma; ATC, anaplastic thyroid carcinoma; RAI-RTC, radioiodine-refractory thyroid cancer; ORR, objective response rate; DLTs, dose-limiting toxicity; SGT, salivary gland tumor; OS, overall survival; PFS, progression-free survival.

#### 2.4.4 Cytotoxic chemotherapy

Chemotherapy is the use of the anticancer or cytotoxic properties of drugs to kill cancer cells. For most malignancies, cytotoxic chemotherapy is a well-documented treatment with good clinical outcomes. The US FDA approved in as early as 1974 doxorubicin for the treatment of DTC, but its clinical benefit is diminished by its toxicity and side effects (180). Therefore, ATA proposed in 2015 that systemic chemotherapy no longer be the standard treatment for RAI-RTCs and only be considered when other options, such as TKIs, are ineffective (3).

#### 2.4.5 Sequential therapy and combined therapy

In addition to the monotherapies adopting the foregoing drugs, sequential therapy and combination therapy also showed certain effects.

The efficacy of sequential therapy was studied in a phase II clinical trial of vemurafenib. Fifty-one patients with *BRAF^V600E^*-positive RAI-RTC were divided into two groups in this trial according to whether they had received VEGFR-targeted TKI treatment before, and both groups were treated with vemurafenib. The antitumor effect was better in patients who had never received TKI therapy (PR, 38.5% vs. 27%; 6-month SD, 35% vs. 27%) (157).

The efficacy of combination therapies has been widely appraised. A retrospective study found that mTOR inhibitor sirolimus combined with chemotherapy drug cyclophosphamide reached a 1-year PFS rate comparable to standard treatment (0.45% vs. 0.30%) in the treatment of RAI-RTCs (181). There is currently a phase II clinical trial in progress that is using the two drugs combination in 19 patients with RAI-RTC (see **Table 2** for details). Temsirolimus, another mTOR inhibitor, in combination with MKI sorafenib achieved PR of 22% and SD of 58% in 36 RAI-RTC patients (182). The combination therapy of somatostatin analog pasireotide and mTOR inhibitor everolimus in the treatment of RAI-RTC was also studied in a phase II clinical trial (NCT01270321), since it was observed that somatostatin receptor (SSTR) 2 was highly expressed in thyroid cancer and activation of the SSTR1-5 inhibited the signal transduction of the PI3K signaling pathway (183). It has been completed, but the results have not been published yet (see **Table 2** for details). Another type of combination therapy is BRAF inhibitor dabrafenib combined with MEK inhibitor trametinib for BRAF-mutated RAI-ATC. In a randomized phase-II open-Label multicenter trial of 53 patients with BRAF-mutated RAI-RTC, this combination therapy was not superior in efficacy compared to dabrafenib monotherapy, within the ORR was 48% versus 42% (184). A new study shown that this combination therapy is effective in BRAF-mutated DTC patients for restoring RAI uptake with PR observed 6 months after RAI administration in 38% of the patients (185). There are also four ongoing clinical trials of dabrafenib in combination with other selective kinase inhibitors (see **Table 2** for details). And two other studies are underway to investigate the response rate of MKI lenvatinib combined with ICIs in patients with RAI-RTC and the reuptake of RAI by lenvatinib on RAI-RTC lesions (see **Table 2** for details).

## 3 Summary and outlook

The in-depth understanding of the molecular mechanisms involved in RAI-RTCs has promoted the development of therapies. Genetic mutations and gene rearrangements at sites such as *RTKs*, *RAS, BRAF* and *TERTp* lead to structural and functional abnormalities of the encoded proteins, which abnormally activate signaling pathways such as the MAPK and PI3K signaling pathways, thus the dedifferentiation of thyroid cells as well as NIS dysfunction, and consequently the RAI-refractory nature of DTCs. Targeted therapy for different mutations provides a new direction for the treatment of RAI-RTCs. The MKI drugs sorafenib, lenvatinib and cabozantinib have been approved by the US FDA for the treatment of RAI-RTCs. As for the other MKIs and selective kinase inhibitors, especially selective kinase inhibitors that restore RAI uptake in tumor cells (such as BRAF, MEK and mTOR inhibitors), encouraging results are shown in multiple clinical trials. However, more multicenter, large-sample, double-blinded randomized controlled trials are anticipated for further verification, and more patients will benefit from the improvement of treatments strategy and the innovation of therapies.
